# MicroRNA Profiling in Mucosal Biopsies of Eosinophilic Esophagitis Patients Pre and Post Treatment with Steroids and Relationship with mRNA Targets

**DOI:** 10.1371/journal.pone.0040676

**Published:** 2012-07-16

**Authors:** Shaolei Lu, Vincent A. Mukkada, Shamlal Mangray, Kelly Cleveland, Nick Shillingford, Christoph Schorl, Alexander S. Brodsky, Murray B. Resnick

**Affiliations:** 1 Department of Pathology and Laboratory Medicine, Rhode Island Hospital, the Alpert School of Medicine, Brown University, Providence, Rhode Island, United States of America; 2 Department of Pediatrics, Hasbro Children’s Hospital, Providence, Rhode Island, United States of America; 3 Department of Molecular Biology, Cell Biology and Biochemistry, Brown University, Providence, Rhode Island, United States of America; South Texas Veterans Health Care System and University Health Science Center San Antonio, United States of America

## Abstract

**Background:**

The characterization of miRNAs and their target mRNAs involved in regulation of the immune process is an area of intense research and relatively little is known governing these processes in allergic inflammation. Here we present novel findings defining the miRNA and mRNA transcriptome in eosinophilic esophagitis (EoE), an increasing recognized allergic disorder.

**Methods:**

Esophageal epithelial miRNA and mRNA from five paired biopsies pre- and post-treatment with glucocorticosteroids were profiled using Taqman and Affymetrix arrays. Validation was performed on additional paired biopsies, untreated EoE specimens and normal controls. Differentially regulated miRNAs and mRNAs were generated, within which miRNA-mRNA target pairs with high predicted confidence were identified.

**Results:**

Compared to the post-glucocorticoid treated esophageal mucosa, of all the 377 miRNA sequences examined, 32 miRNAs were significantly upregulated and four downregulated in the pre-treated biopsies. MiR-214 was the most upregulated (150 fold) and miR-146b-5b, 146a, 145, 142-3p and 21 were upregulated by at least 10 fold. Out of 12 miRNAs chosen for validation by qRT-PCR, five (miR-214, 146b-5p, 146a, 142-3p and 21) were confirmed and 11 shared the same trend. When the expression of the 12 miRNAs in the EoE mucosa was compared to unrelated normal mucosa, six (miR-214, 146b-5p, 146a, 21, 203, and 489) showed similar significant changes as in the paired samples and 10 of them shared the same trend. In the same five pairs of samples used to profile miRNA, 311 mRNAs were down-regulated and 35 were up-regulated in pre-treated EoE mucosa. Among them, 164 mRNAs were identified as potential targets of differentially regulated miRNAs. Further analysis revealed that immune-related genes, targeted and non-targeted by miRNAs, were among the most important genes involved in the pathogenesis of EoE.

**Conclusions:**

Our findings add to the accumulating body of data defining a regulatory role for miRNA in immune and allergic processes.

## Introduction

Eosinophilic esophagitis (EoE) is an increasingly recognized antigen-driven disorder of the esophagus occurring in children and adults [1]. Diagnosis of the disorder is based on clinicopathological correlation between the patient’s clinical manifestations and histologic findings on mucosal biopsies. There is increasing evidence to support that EoE can be an aero-and food allergen driven process in which Th2 derived cytokines and chemokines play important roles [2]. Recently published studies have focused on the EoE transcriptome and a number of dysregulated genes influencing key cellular players such as eosinophils, lymphocytes, mast cells, esophageal epithelial cells and subepithelial myofibroblasts have been characterized [3], [4].

MicroRNAs (miRNAs) are small, non-coding 19–25 nucleotide long RNAs that constitute the most abundant class of regulators of gene expression [5]. Their primary mechanism of action is post-transcriptional regulation via RNA interference leading to mRNA strand degradation or translational inhibition. MiRNAs participate in the regulation of a wide range of physiological and pathological processes including development, neoplasia, stress response and inflammation. The interplay between miRNA and mRNA is complex as each mammalian genome contains several hundred miRNAs, a single miRNA may regulate several mRNAs and each mRNA may be affected by several different miRNAs [6].

The characterization of miRNAs and their target mRNAs involved in regulation of the immune process is an area of intense research [7]. Although a number of studies have described the miRNA profile in other allergic disorders [8]–[12], only one recent study addressed the miRNA profile of EoE esophageal mucosa [13].

This goal of this study was to characterize the miRNA profile of well documented EoE mucosal biopsies, before and after successful treatment with glucocorticosteroids, and to correlate this profile with dysregulated mRNA identified using the same cohort. These findings were further verified on an additional cohort of biopsies from patients with EoE as opposed to normal mucosa.

## Materials and Methods

### Patients and Biopsies

Archival esophageal biopsies from seven pediatric patients before and after treatment for EoE were retrieved from the Department of Pathology at Rhode Island Hospital (Providence, RI). The first five pairs were used for both miRNA and mRNA profiling and all seven were used for validation (Table 1). Eight additional samples from patients newly diagnosed with EoE (from whom no post-treatment sample was available for analysis) and 10 control samples from pediatric patients with normal esophageal mucosa were also obtained (Table 1).

**Table 1:** Clinical features of the eosinophilic esophagitis patients.

Patient Group	No.	Age at Diagnosis	Gender	Presenting Complaint	Food Allergy	Steroid Used
Paird EoE patients	1	11	M	Ab pain	Yes	Bud
	2	5	M	Vomiting, Food refusal	Yes	Bud
	3	2	M	Regurgitation	Yes	Bud
	4	13	F	Dysphagia, Chest pain	Yes	Bud
	5	2	F	Vomiting, FTT	No	Bud
	6	9	M	Vomiting, Food refusal	No	Flu
	7	1.3	M	FTT, Food refusal	Yes	Flu
Untreated EoE	1	5	M	Dysphagia	Yes	
	2	16	M	Dysphagia	Yes	
	3	10	M	Dysphagia, Reflux symptoms	No	
	4	2	M	Food refusal	Yes	
	5	10	M	Reflux symptoms, Ab pain	No	
	6	1.5	M	Dysphagia	Yes	
	7	15	F	Diarrhea	No	
	8	14	F	Ab Pain, Diarrhea	No	
Normal Control	1	13	F	Ab pain, Diarrhea, Wt loss	No	
	2	14	F	Ab pain, Fatigue	No	
	3	15	F	Ab pain	No	
	4	5	M	Ab Pain	No	
	5	6.5	M	Dysphagia,	Yes	
	6	17	F	Nausea	Yes	
	7	17	F	Hematochezia	No	
	8	3	F	Food refusal, Wt loss	No	
	9	16	M	Ab pain	No	
	10	8	F	Ab pain	No	

Abbreviations used: Ab, Abdominal; Wt, weight; FTT, Failure to Thrive; Bud, Budesonide; Flu, Fluticasone.

Patient records were reviewed by a pediatric gastroenterologist (V.M.) to ensure that the clinical characteristics of the patients fit the diagnosis of EoE. The slides were reviewed by a pathologist (M.R.) to confirm the presence or absence of EoE. Classic features of EoE consist of numerous intraepithelial eosinophils greater than 15 per high powered field (HPF) (ranging from 35 to over 100 per HPF), superficial eosinophilic microabscesses, basal layer hyperplasia and subepithelial fibrosis. The posttreatment biopsy material consisted of normal appearing squamous mucosa with only rare intraepithelial eosinophils numbering less than 2 per HPF.

A summary of the seven EoE patients’ clinical information is presented in Table 1. Of the seven EoE patients evaluated prior to and following corticosteroid therapy five were males and two were females ranging from 16 months of age through 12 years. The presenting symptoms were a combination of abdominal pain, vomiting, dysphagia and chest pain in the older children and vomiting, food refusal and failure to thrive in the younger patients. Five of the seven patients tested positive for other food allergies. All of the patients responded completely to either budesonide or fluticasone.

The study was performed according to a protocol approved by the institutional review board (IRB) of Lifespan/Rhode Island Hospital. The requirement of consent was specifically waived by the IRB committee.

### RNA Extraction and Quality Assessment

Eight 10 µm sections were cut from each block and mounted onto plain glass slides. If the biopsy section contained only epithelium, the sections were scraped from the slides and were ready for total RNA extraction. If biopsy sections contained sub-epithelium, the sections were deparaffinized, stained, dehydrated through graded alcohols using the Paradise FFPE reagent System (Applied Biosystems, Foster City, CA) and subjected to LCM within 2 hours of deparaffinization. About 20,000 epithelial cells were captured on LCM Macro CapSure caps (Applied Biosystems) using the Arcturus XT LCM instrument (Applied Biosystems) and the captured cells in the caps were used to extract total RNA. In either case, only the RNA from the epithelial mucosa was extracted. RecoverAll Total Nucleic Acid Extraction Kits for FFPE tissues (Ambion, Austin, TX) were used for extraction. RNA was further purified and concentrated using the RNEasy Minelute Cleanup Kit (Qiagen, Valencia, CA), and then evaluated by the Agilent Bioanalyzer using an RNA 6000 Nano or Pico LabChip (Agilent Technologies Santa Clara, CA) as described previously [14].

### MicroRNA Expression Analysis by Taqman Low-density Array

Five nanograms of total RNA was reverse-transcribed using the Taqman MicroRNA Reverse Transcription Kit and the Megaplex RT primer Human Pool A (Applied Biosystems). The reverse-transcribed cDNA was then pre-amplified in 12 cycles of PCR using Taqman PreAmp Master Mix and the Megaplex PreAmp primers, Human Pool A (Applied Biosystems). The cDNAs were then diluted and loaded onto a Taqman Human miRNA Array card A (Applied Biosystems), which contains probes for 377 distinct miRNAs and a housekeeping gene (MammU6). The Array cards were run on an ABI HT7900 quantitative PCR (qPCR) instrument. Ct values were obtained for all miRNAs represented on the cards and fold changes in expression were calculated using the delta delta Ct (ddCt) method. Expression levels of MammU6 on the Array card were used as control for the purpose of ddCt calculation. The expression array data have been deposited in Gene Expression Omnibus (GEO) of National Center for Biotechnology Information (NCBI) and are accessible through GEO series accession number GSE36726.

### Confirmation of miRNA Expression Data by qRT-PCR

Expression of miRNAs that demonstrated high differential expression by array card in five matched EoE cases before and after treatment were confirmed again in those same matched cases as well as in two additional matched cases. In addition, the expression of these differentially expressed miRNAs was determined in eight new samples of EoE biopsy material and compared to ten biopsies from unrelated normal esophageal epithelium (Table 1). Five nanograms of total RNA was reverse transcribed using the Taqman MicroRNA Reverse Transcription Kit and a pool of specific RT primers for the 12 miRNAs chosen for confirmation and a housekeeping gene (MammU6) (Applied Biosystems). The RT products were then pre-amplified using the same pool of the PCR primers and the pre-amplified products were used as templates for the individual Taqman miRNA assays which were run on an Agilent MX3005p qPCR instrument. Fold changes were again calculated using the ddCt method.

### Amplification of Total RNA for mRNA Expression Analysis by Affymetrix Expression Array and qRT-PCR

Fifty nanograms of total RNA was amplified and reverse-transcribed into cDNA using Ovation FFPE WTA system (Nugen Technologies, San Carlos, CA). Five to ten micrograms of amplified cDNA from the amplification above was fragmented and labeled for Affymetrix array analysis using Encore Biotin Module (Nugen Technologies). Array hybridization and analysis were performed by Genomics Core Facility at the Center for Genomics and Proteomics (Brown University, Providence, RI) according to Affymetrix protocols (Affymetrix Inc, Santa Clara, CA).

### Bioinformatic Data Analysis

Affymetrix Gene 1.0 ST Array data was analyzed by Genomics Core Facility using Affymetrix Command Console and Expression Control. The expression array data have been deposited in GEO of NCBI and are accessible through GEO series accession number GSE36725.

Functional annotation was performed using Partek Genomics Suite 6.0, Partek Inc, St. Louis, MI). Differentially regulated genes are selected based on the fold-change (FC), false discovery rate (FDR), and statistical significance (P-value of Student’s paired t-test). Target genes for miRNA were predicted using Ingenuity Pathway Analysis (IPA, Ingenuity Systems, Inc. Redwood City, CA). The miRNA and mRNA in a pair have to be regulated in opposite directions and their interactions are either “Experimentally observed” or exhibit “High (predicted) Confidence” as defined by IPA. Functional pathway analysis and network analysis were performed using modules within IPA.

## Results

### Identification of EoE Specific miRNA Transcripts

**Figure 1:**
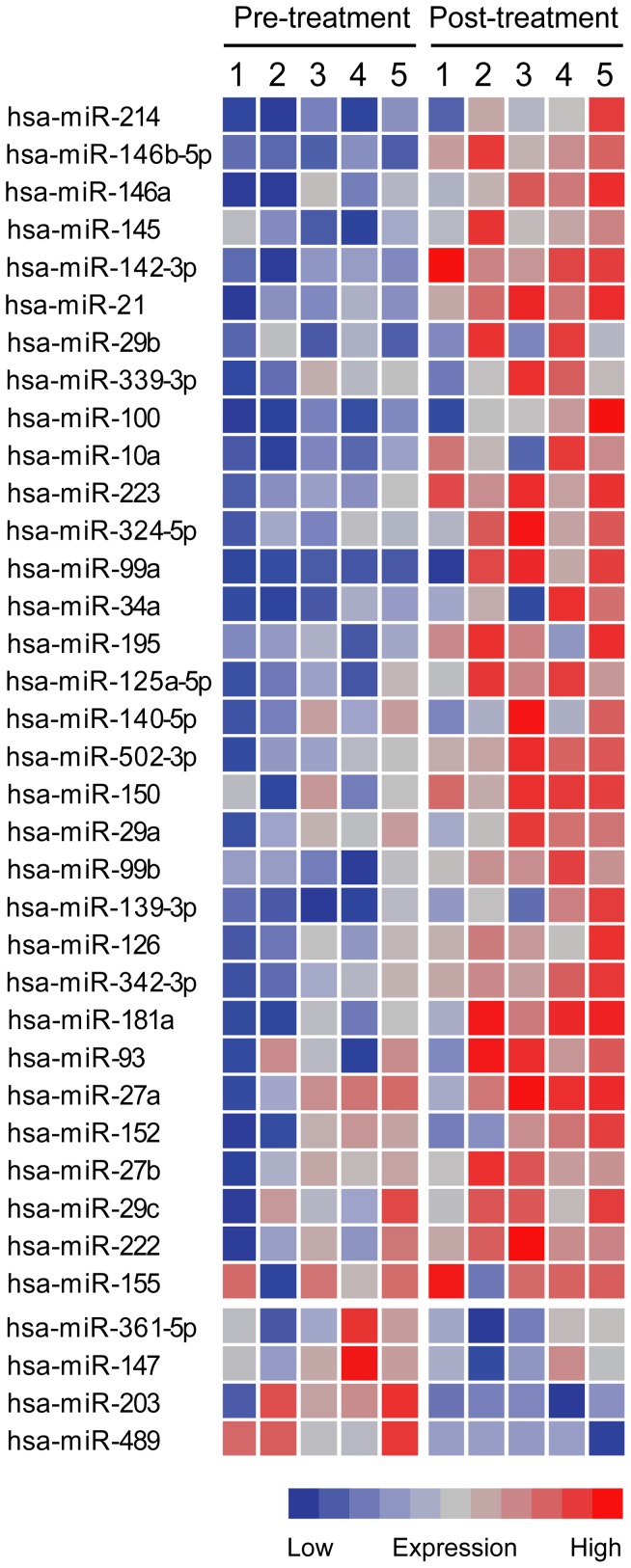
Steroid treatment regulates miRNA expression of EoE mucosa. The heat map illustrates miRNA expression in five paired esophageal epithelium samples of EoE purified before and after treatment. Each column represents one sample. MiRNA expression levels were stratified individually for each miRNA and each square was colorized based on the level of its expression. The overall fold changes pre- and post-treatment are listed in Table 2.

Total RNA from five pairs of EoE biospies before and after treatment was extracted and miRNA expression levels were quantified by Taqman Low Density Array. Of all the miRNAs tested by the array, 36 of them were differentially regulated based on a paired t-test P-value of less than 0.05. Five transcripts were down-regulated after treatment and 33 were up-regulated. The absolute fold changes range from 1.6 to 151. Some miRNAs share the same target sites so that a total of 31 target sites were identified from all the miRNAs. MiR-373 and miR-520e share the same target sites while miR-373 is up-regulated and miR-520e is down-regulated post treatment and thus are excluded for further analysis. The differentially regulated miRNAs are listed in Table 2, and a heatmap is presented in Figure 1 which includes 36 miRNAs and 30 target sites.

**Table 2:** Taqman Array data of differentially regulated miRNAs in esophageal epithelium of EoE patients before vs following treatment with glucocorticosteroids.

miRNA ID	Overlapping target sites	P-value*	Fold change**
hsa-miR-214		0.019	151.46
hsa-miR-146b-5p		0.0015	47.15
hsa-miR-146a	Same as miR-146b-5p	0.00045	39.22
hsa-miR-145		0.028	35.95
hsa-miR-142-3p		0.0017	26.55
hsa-miR-21		0.0010	11.51
hsa-miR-29b		0.015	9.43
hsa-miR-339-3p		0.018	7.79
hsa-miR-100		0.025	7.27
hsa-miR-10a		0.048	6.70
hsa-miR-223		0.0028	5.44
hsa-miR-324-5p		0.014	5.34
hsa-miR-99a	Same as miR-100	0.024	5.20
hsa-miR-34a		0.030	4.99
hsa-miR-195		0.0041	4.87
hsa-miR-125a-5p		0.019	4.75
hsa-miR-140-5p		0.017	4.64
hsa-miR-502-3p		0.0020	4.62
hsa-miR-150		0.0043	4.53
hsa-miR-29a	Same as miR-29b	0.0060	4.28
hsa-miR-99b	Same as miR-100	0.033	4.13
hsa-miR-139-3p		0.020	3.86
hsa-miR-126		0.010	3.82
hsa-miR-342-3p		0.0019	3.22
hsa-miR-181a		0.012	3.19
hsa-miR-93		0.019	2.95
hsa-miR-27a		0.0038	2.93
hsa-miR-152		0.0058	2.87
hsa-miR-27b	Same as miR-27a	0.043	2.05
hsa-miR-29c	Same as miR-29b	0.036	1.97
hsa-miR-222		0.026	1.85
hsa-miR-155		0.035	1.58
hsa-miR-361-5p		0.038	−2.22
hsa-miR-147		0.0064	−2.45
hsa-miR-203		0.036	−2.68
hsa-miR-489		0.037	−2.76

* Paired Student’s T-test; comparing the expression levels before and after treatment.

** Fold change  =  expression levels before/after treatment.

### Confirmation of Individual miRNA Expression by Quantitative RT-PCR in Additional EoE Biopsies

**Figure 2:**
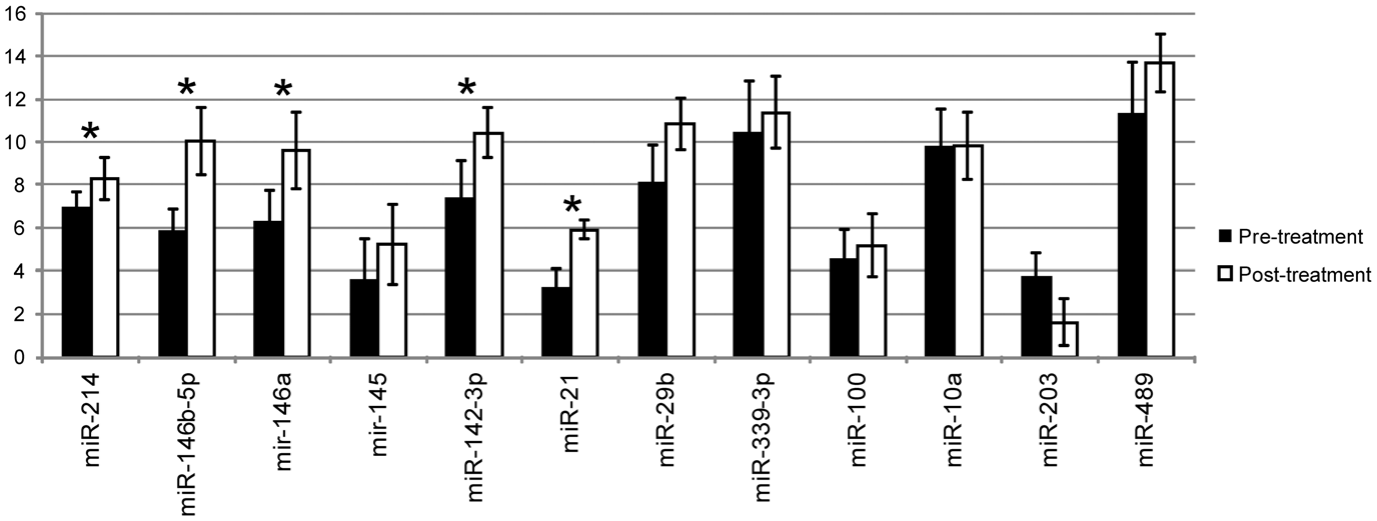
Treatment-induced miRNA expression changes validated by qRT-PCR. Delta Ct values are used to represent selected miRNA expression levels in esophageal epithelium of 7 paired EoE patients pre- vs post-treatment (5 pairs used in miRNA profiling and 2 additional pairs; Table 1). Asterisks indicate statistical significance (P<0.05).

To validate the expression of the differentially regulated miRNAs discovered by Taqman Low Density Array in Table 2, the same five paired biopsy specimens from the previous section and two additional paired biopsy specimens for a total of seven samples before and after treatment were evaluated by qRT-PCR. Twelve miRNAs that demonstrated high differential expression by Array card (the top ten upregulated and the bottom two downregulated) were chosen for validation by qRT-PCR. Five miRNAs (miR-214, miR-146b-5p, miR-146a, miR-142-3p and miR-21) were confirmed with statistical significance (P-value <0.05) and 11 of the miRNAs (except miR489) shared the same regulatory trends (Table 3 and Figure 2).

**Table 3:** qRT-PCR validation of differentially regulated miRNAs in esophageal epithelium of EoE patients pre-vs post-treatment and untreated EoE patients vs normal control.

			qRT-PCR Verification			
miRNA	Taqman Array		Pre- vs post treatment		EoE vs Normal	
	Fold Change	p-value	Fold Change	p-value	Fold Change	p-value
hsa-miR-214	151.45	0.019	26.62	0.02	2.02	0.078
hsa-miR-146b-5p	47.15	0.0015	18.79	0.0031	5.4	0.004
hsa-miR-146a	39.22	0.00045	9.89	0.0022	3.1	0.033
hsa-miR-145	35.95	0.028	3.15	0.079	1.73	0.1
hsa-miR-142-3p	26.55	0.0017	8.61	0.019	1.3	0.12
hsa-miR-21	11.51	0.001	6.67	0.006	4.39	0.003
hsa-miR-29b	9.43	0.015	13.04	0.09	0.97	0.24
hsa-miR-339-3p	7.78	0.018	1.92	0.31	1.21	0.28
hsa-miR-100	7.27	0.025	1.58	0.38	0.93	0.25
hsa-miR-10a	6.7	0.048	1.03	0.49	0.38	0.062
hsa-miR-203	0.37	0.036	0.237	0.072	0.19	0.0006
hsa-miR-489	0.36	0.037	5.27	0.21	0.13	0.042

**Figure 3:**
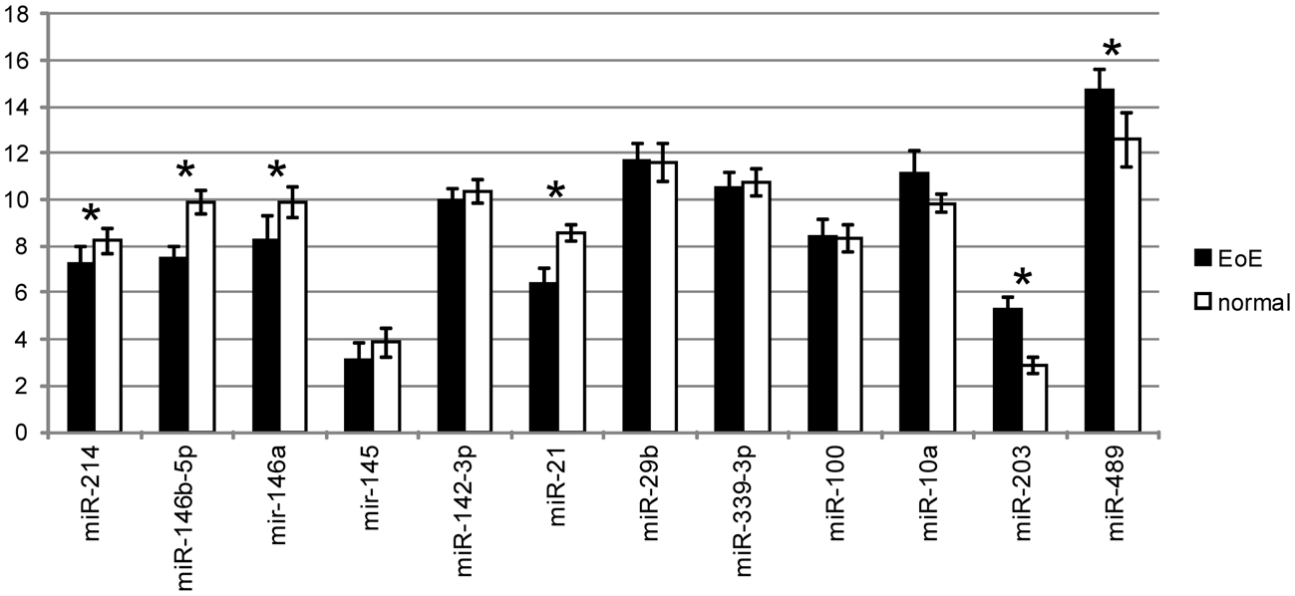
EoE-related miRNA expression changes determined by qRT-PCR. Delta Ct values are used to represent selected miRNAs expression levels in esophageal epithelium of 8 untreated EoE patients versus 10 normal epithelium of control patients (Table 1). Asterisks indicate statistical significance (P<0.05).

Paired biopsies from the same patient were used to profile the miRNA expression for the purpose of eliminating the inter-individual variation. However, as glucocorticosteroids were the major treatment component in our patient’s cohort, miRNA expression following treatment may be related in part to the treatment effect. To test this hypothesis, we compared the miRNA expression of the mucosal biopsies from a new set of eight EoE patients with those from normal mucosa of ten additional pediatric esophageal biopsy samples from patients without any history of EoE. As shown in Table 3 and Figure 3, six out of same 12 miRNAs (miR-214, miR-146b-5p, miR-146a, miR-21, miR-203, and miR489) showed similar significant differential expression as in the paired samples and 10 of them (except for miR-10a and miR-29b) shared the same regulatory trends.

### Bioinformatic Analysis of miRNA Targets

**Figure 4:**
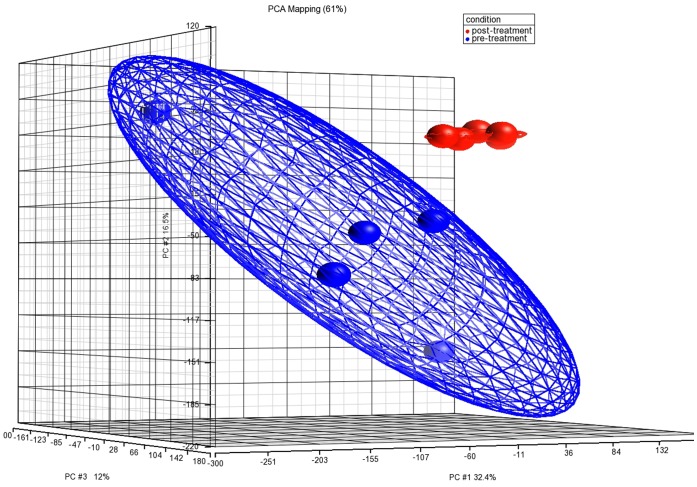
Principle component analysis of mRNA expression before and after treatment of EoE. In this 3-dimentional plot of the first three principle components of all ten samples, samples of the same treatment status were grouped together. Five pre-treatment samples (Blue) and five post-treatment samples (Red) were clearly separated from each other.

The major role of miRNA is to regulate mRNA expression at the transcriptional and translational level. We wished to determine how much of the mRNA expression in EoE is influenced by miRNAs. To profile the global mRNA changes in EoE, an Affymetrix GeneChip based mRNA gene profiling was conducted on five paired EoE biopsies before and after treatment. Total RNA was extracted from FFPE tissue sections and hybridized to Human Gene ST 1.0 Genechips. The Principle Component Analysis of data demonstrated that gene expression data from biopsies of the same status are highly correlated with each other (Figure 4). To screen for differentially regulated genes, FC >2, FDR<0.05 and P-value <0.05 were used as criteria. Of all the probes on the chip, 415 probes passed the screen resulting in 346 differentially regulated genes, of which 311 genes were down-regulated and 35 genes were up-regulated in EoE. Among them, 164 genes were identified as potential targets of miRNAs listed in Table 2 under stringent conditions using Ingenuity Pathway Analysis. Table 4 lists the mRNAs with the highest fold change for each miRNA. Many of the mRNAs can be targeted by more than one miRNA. A full list of all differentially regulated mRNAs regardless of fold change is available in Table S1. Of these mRNAs, 162 were down-regulated and 2 were up-regulated. The remaining 184 genes out of 346 differentially regulated genes are not targeted by miRNA.

**Table 4:** A total of 164 mRNAs targeted by 31 miRNAs (25 target sites)* differentially regulated in EoE epithelium.

miRNAs and Target mRNAs	Fold Change
**hsa-miR-203**	−**2.68**
ALOX15	10.30
**hsa-miR-147**	−**2.45**
CYP2S1,	2.03
**hsa-miR-10a**	**6.70**
ZFAND5, CNST, RAP2A, ACTG1, TIAM1	−2.78 ∼ −2.00
**hsa-miR-125a-5p**	**4.75**
ETNK2, IL1RN, PRDM1, B4GALT1, GRHL1, TRPS1, FAM129B, RIT1, DOCK3, MEGF9	−4.88 ∼ −2.08
**hsa-miR-126**	**3.83**
TOM1	2.67
**hsa-miR-140-5p**	**4.64**
PHACTR2, STRADB, SNX16, RALA, RIOK3, C15orf29, PRDM1, MYO6, CAMK2N1, MRPS10	−4.49 ∼ −2.24
**hsa-miR-142-3p**	**26.55**
MGLL, C18orf25, USP6NL, CCDC6, RAB2A, TIPARP, HECTD1, KAT2B, WASL, TWF1, C1orf9	−3.00 ∼ −2.00
**hsa-miR-145**	**35.95**
PADI1, BNIP3, PHACTR2, SNX24, KIF21A, TSPAN6, SAMD5, SRGAP1, TMOD3, AIM1	−6.97 ∼ −2.99
**hsa-miR-146a and miR-146b-5p**	**47.15 ∼ 39.22**
S100A12, ZNF117, MYO6, CCDC6, IL1RAP, IL1RL2	−6.70 ∼ −2.1
**hsa-miR-150**	**4.53**
SYNPO2L, PDIA6, EREG, TOM1, CNST, TRPS1, ADIPOR2, CAST, AIFM2, FOPNL	−3.63 ∼ −2.20
**hsa-miR-152**	**2.87**
RMND5A, PHACTR2, EMP1, MXD1, STRADB, B4GALT5, MOSPD1, C18orf25, CLOCK, KIAA0232	−4.82 ∼ −2.61
**hsa-miR-155**	**1.58**
ETNK2, ZNF431, PPL, WNK1, TRPS1, CAB39, PEA15, PTN, ZNF714, TWF1	−4.88 ∼ −2.03
**hsa-miR-181a**	**3.19**
PHACTR2, SASH1, PHLDA1, MOSPD1, SRGAP1, C12orf29, UBL3, PHACTR4, EREG, RALA	−4.49 ∼ −2.83
**hsa-miR-195**	**4.87**
ALOX12, PHACTR2, CGNL1, KIF21A, STRADB, RASGEF1B, TUFT1, PDIA6, SNX16, TRIP10	−8.65 ∼ −2.92
**hsa-miR-21**	**11.51**
IL12A, RMND5A, SASH1, PELI1, TOR1AIP2, SECISBP2L, HIPK3, RFFL, TIAM1	−6.25 ∼ −2.00
**hsa-miR-214**	**151.46**
ABLIM3, DOCK9, ARHGAP10, PIM1, TRPS1, CPEB4, RAB5B, CAPN5, TWF1, KIF1B	−5.08 ∼ −2.01
**hsa-miR-222**	**1.85**
PHACTR4, C18orf25, INPP4B, ZFAND5, KIAA1370, TRPS1, LYPLA1, TIPARP, TP53BP2, MEGF9	−2.89 ∼ −2.08
**hsa-miR-223**	**5.44**
NAMPT, SNX24, MSMO1, PRDM1, CDS1, SECISBP2L, TWF1, ZNF365	−4.01 ∼ −2.02
**hsa-miR-27a and miR-27b**	**2.94 ∼ 2.05**
ENDOU, BNIP3, RMND5A, CRISP2, LPIN1, CCNYL1, CAB39L, CPPED1, CNN3, WNK1	−6.94 ∼ −2.81
**hsa-miR-29a, miR-29b and miR-29c**	**9.43 ∼ 4.28**
PHACTR2, TUBB2A, EMP1, SNX24, MXD1, AMFR, AIM1, RIOK3, WDR26, DSC2	−4.49 ∼ −2,53
**hsa-miR-342-3p**	**3.22**
RMND5A, SYNPO2L, CAMK2N1, CPEB4, C20orf11, TIAM1	−7.19 ∼ −2.00
**hsa-miR-34a**	**4.99**
ABLIM1,TOM1,CLOCK,NCOA1,AREG, PEA15, MARCH5, DOCK3,RNF169, B4GALR5, AIM1	−2.92 ∼ −2.99
**hsa-miR-502-3p**	**4.62**
B4GALT5,AIM1	−3.36 ∼ −2.99
**hsa-miR-93**	**2.95**
MXD1, SASH1, NPAS2, HIF1A, SLC16A9, MSMO1, SNX9, MGLL, SNX16, TRIP10, EREG	−3.96 ∼ −2.99
**hsa-miR-99a, miR-9b and miR-100**	**5.20**
GRHL1, PPP1CB	−2.58 ∼ −2.18

* Only up to 10 mRNAs with the highest fold change are listed under each miRNA.

** miR-339-3p, miR-489, miR-361-5p, miR-324-5p, and miR-139-3p do not have predicted targets in combined gene list.

**Figure 5:**
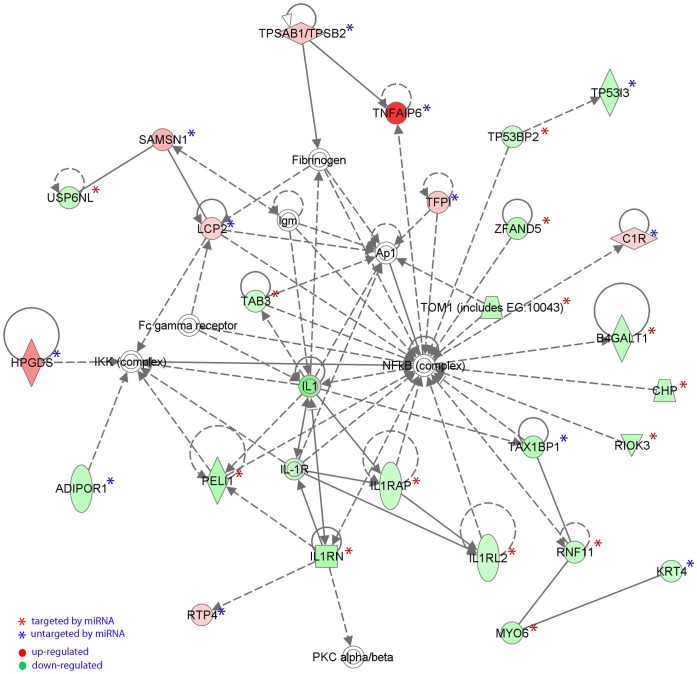
Functional pathway network analysis of 346 differentially regulated mRNAs. One of the primary pathway networks is associated with network functions of “Inflammatory Response, Cell-To-Cell Signaling and Interaction, Hematological System Development and Function”.

Information regarding the miRNA-mRNA pairs is incorporated into a pathway network analysis on the 346 differentially regulated genes using IPA program. One of the top pathway networks IPA revealed is a gene regulatory network of more than 24 genes involving “inflammatory response”, “cell-to-cell signalling and interaction”, and “hematological system development and function” (Figure 5). Within this network 14 genes are targeted by miRNAs while 11 are not targeted. Interestingly genes involving the IL1 cytokine pathway are exclusively targeted by miRNAs (Figure 5).

Given the finding that both targeted genes and non-targeted genes are equally represented in the network (Figure 5), we conducted a full search of immune-related genes in the 346 differentially regulated genes. Indeed, immune-related genes were identified in both groups. Out of 164 targeted genes, 69 of them are related to the immune process, of which one gene was up-regulated and the rest were down-regulated (Table 5). Out of 184 untargeted mRNAs, 59 are immune related genes (Table 6).

**Table 5:** List of 69 Immune related genes targeted by differentially regulated miRNAs in EoE epithelium.

ID	p-value	Fold Change	ID	p-value	Fold Change
ALOX15	0.0012	10.30	B4GALT1	0.00098	−2.59
ALOX12	3.4E-05	−8.65	NCOA1	0.00044	-2.56
S100A12	0.00014	−6.70	DSC2	0.00047	−2.53
IL12A	5.0E-05	−6.25	PIM1	4.3E-05	−2.44
ETNK2	7.7E-05	−4.89	CNST	0.00021	−2.42
LPIN1	6.4E-05	−4.60	KIAA1370	0.00038	−2.41
PHACTR2	4.7E-05	−4.49	TRPS1	0.00013	−2.39
TUBB2A	0.00036	−4.19	CCDC6	1.9E-05	−2.38
NAMPT	0.0011	−4.01	ADIPOR2	0.00042	−2.35
SNX24	0.00017	−4.00	PSD3	0.00049	−2.35
MXD1	0.0012	−3.96	CPEB4	0.00092	−2.33
NPAS2	8.7E-05	−3.58	CYP4F3	1.2E-05	−2.35
CPPED1	0.00096	−3.53	AREG	0.0004	−2.32
HIF1A	0.00020	−3.45	CAST	0.0012	−2.31
SC4MOL	0.00023	−3.33	RAB2A	5.1E-05	−2.30
SAMD5	0.00028	−3.24	ACTG1	0.00027	−2.22
IL1RN	0.00026	−3.16	GNG12	3.8E-05	−2.20
AMFR	2.6E-06	−3.13	PPP1CB	0.00078	−2.18
PELI1	0.00033	−3.13	EPB41L4A	1.7E-06	−2.15
CNN3	0.00064	−3.09	MARCH5	0.00096	−2.14
PDIA6	0.00037	−3.01	DOCK3	0.00024	−2.14
MGLL	6.8E-07	−3.00	WASL	0.00025	−2.12
ABLIM1	4.2E-05	−2.92	GABARAPL2	0.00037	−2.11
UBL3	0.00044	−2.90	IL1RAP	8.0E-05	−2.10
EREG	0.00014	−2.89	RAB5B	0.00077	−2.10
C18orf25	0.00063	−2.86	STK39	0.0011	−2.08
PFKFB3	7.6E-05	−2.84	ZNF714	0.00096	−2.08
INPP4B	0.00014	−2.84	PI4K2A	0.00047	−2.06
RALA	0.00070	−2.83	PLEKHA6	0.00013	−2.06
WNK1	5.16E-06	−2.81	ZNF365	0.00035	−2.02
KRT7	8.21E-06	−2.69	KIF1B	0.00051	−2.01
PLEKHM1	0.00025	−2.69	RNF169	0.00080	−2.00
RIOK3	0.00038	−2.65	IL13RA1	0.0010	−2.00
CLOCK	0.00089	−2.62	TIAM1	8.0E-05	−2.00
PRDM1	0.00063	−2.59			

* Genes related to “hypersensitivity response”, “immunological disease”, “inflammatory disease”, and “inflammatory response” by IPA annotation are selected to represent immune related genes.

**Table 6:** List of 59 Immune related genes not targeted by differentially regulated miRNAs in the esophageal epithelium of EoE patients.

ID	p-value	Fold Change	ID	p-value	Fold Change
TNFAIP6	0.00033	39.23	WNT5A	0.00099	−3.32
CPA3	7.0E-05	7.70	SCNN1B	5.2E-05	−3.28
HPGDS	3.0E-05	5.87	TMEM57	0.00011	−3.17
SAMSN1	0.00017	3.82	GNG4	0.0011	−2.97
SERPINE2	0.00046	3.15	TAX1BP1	0.00025	−2.94
TPSAB1	0.00069	3.02	MAPK3	1.4E-05	−2.92
TFPI	0.00019	2.82	UACA	0.00011	−2.81
LAPTM5	0.00091	2.6	ZNF92	9.2E-06	−2.63
LCP2	0.00016	2.41	ST3GAL4	1.5E-06	−2.62
CNTN4	0.00020	2.30	ADIPOR1	0.00015	−2.58
HIST1H4K	0.00077	2.24	EFNA5	0.00076	−2.57
C1R	0.00039	2.23	ZDHHC21	0.0012	−2.54
HDC	0.00025	2.21	S100A9	0.00089	−2.36
IGFBP3	0.00097	2.18	NHSL1	0.00016	−2.34
LOX	0.00016	2.15	DOPEY2	0.00031	−2.33
B2M	0.00088	2.08	CD68	0.00046	−2.29
IFITM3	0.00043	2.03	GNA15	0.00041	−2.22
SLURP1	0.000062	−8.13	DYNLL1	0.00024	−2.21
SFTA2	0.00011	−7.87	S100A14	6.6E-05	−2.18
CRYAB	0.00032	−5.74	DSG1	0.00034	−2.15
IL18	0.00015	−5.11	AHCY	0.00036	−2.15
CST6	0.0012	−4.25	RALB	0.00038	−2.14
PSCA	0.00029	−4.14	GPT2	0.0011	−2.12
CXCR2	0.000053	−4.12	PARD3	0.00013	−2.12
ACPP	0.00017	−3.98	LGMN	0.00082	−2.11
C3orf67	1.9E-07	−3.90	PLD1	0.00055	−2.08
CDA	0.001	−3.71	PCSK5	0.00071	−2.08
RHCG	0.00033	−3.7	OSTF1	0.00026	−2.04
MTHFD1L	0.00092	−3.51	CDK7	0.00082	−2.01
TPRG1	0.00081	−3.43			

* Genes related to “hypersensitivity response”, “immunological disease”, “inflammatory disease”, and “inflammatory response” by IPA annotation are selected to represent immune related genes.

## Discussion

Little is known regarding miRNA regulatory pathways in allergic inflammation in general [8]–[12] and this is the second report to address differential miRNA expression in EoE [13]. This study is unique in that it correlated miRNA and mRNA expression in the same patient population before and after treatment with glucocorticosteroids, the tissue sampling was restricted to the epithelial layer and it was performed on archival formalin-fixed paraffin embedded tissues.

In our study 36 miRNAs were differentially expressed (32 upregulated and 4 downregulated) in EoE before and following treatment including 25 miRNAs at a level of 3 fold or more. Differential miRNA expression was correlated with differential mRNA expression in the same patients: 311 genes were found to be downregulated and 35 upregulated when comparing the pretreated to posttreated samples. Several of the mRNAs identified have been shown to play a role in EoE or in other allergic disorders. In general the results of this study are more heavily weighted towards miRNAs with increased expression levels and mRNAs with decreased expression levels in the inflammatory state as opposed to the treated samples. The opposing direction of the miRNA and mRNA expression is in line with miRNA’s regulatory role on gene expression [5].

In an attempt to better define how much of the differential regulation of miRNAs in EoE was related to the effect of the glucocorticosteroid treatment on the allergic process as opposed to the allergic process alone we examined the expression levels of the miRNAs originally detected to those in additional material from EoE patients compared to control tissue from histologically normal appearing esophageal mucosa. The fold changes as detected by qPCR were significantly higher in the pre vs posttreatment tissue than in the EoE vs control tissue and not all of the qRT-PCR differential expression reached statistical significance. The differential expression of certain of these miRNAs such as miR-214, miR-142-3p and miR-29b appear to be influenced much more significantly by the treatment as opposed to the allergic process alone.

Another important issue is to determine which cell types are responsible for the differential expression of miRNAs in this study. We purposely dissected areas of epithelium only and avoided submucosal stromal tissue in many cases employing LCM for this purpose. In addition to eosinophils the squamous epithelium in EoE also exhibits increased infiltration by T-cells and mast cells [2]. Based on a recent study by Allantaz et al which determined the expression profile of human immune subsets, the only miRNA that was differentially regulated in our study and found to be differentially regulated by immune cells was miR-223 [15]. In Allantaz’s study miR-223 was overexpressed in neutrophils, eosinophils and monocytes [15] whereas in our study there was a five fold increase in biopsies of EoE patients prior to treatment. Thus one may speculate that increased miR-223 in EoE is due to the intraepithelial inflammatory population. Interestingly miR-223 was also shown to be overexpressed in squamous cell carcinomas of the esophagus suggesting that the increased epithelial proliferation characteristic of EoE may also be a contributor to the overexpression of miR-223 [16]. Unfortunately although differential miRNA expression is an area of significant ongoing research in neoplastic processes of the esophagus [16]–[17] little is known regarding baseline expression of miRNAs by normal squamous epithelium or by actively proliferating esophageal epithelium characteristic of EoE.

A number of miRNAs that were found to be differentially regulated in this study were implicated by others to play a role in allergic and inflammatory disorders [8]–[13]. There is considerable overlap between the miRNAs that we identified here and those published in the recent report by Lu et al [13], however, a number of differentially miRNAs such as miR-214, miR-145, miR-100 and miR10a were uniquely identified in our study. Furthermore miR-214 was the most significantly elevated miRNA upregulated in EoE patients before treatment. These differences may be attributed to the fact that our study was restricted to harvesting RNA from the epithelial layer alone, at times employing LCM. The submucosal esophageal tissue of EoE patients is rich in inflammatory cells and myofibroblasts thus diluting the influence of epithelial associated mRNA and miRNA. Our study was also based on material that was paraffin embedded and formalin fixed and the initial screen was performed on pretreated vs post-treated biopsy material from the same patient to avoid the variability of comparing pathological material from one source to control tissue from a different source.

Although miR-214 has been implicated in a number of neoplastic disorders, to date a role for miR-214 in inflammatory or allergic disorders has not been described. MiR-214 has been found to be elevated in models of renal injury and may play a role in epithelial mesenchymal transition [18], [19]. The epithelial mesenchymal transformation (EMT) pathway likely plays an important role in the subepithelial fibrosis characteristic of airway disease in asthma [20], [21]. This pathway might also be involved in the generation of subepithelial fibrosis typically seen in EoE [22]–[24]. A recent report by Kagawalla et al supports a role for EMT in EoE [25].

Both miR-146a and miR-146b were markedly increased in the epithelium of EoE patients. MiR-146a has been shown to regulate several physiological and pathophysiological pathways in hematopoietic cells as reviewed by Hua et al [26]. MiR-146a has been shown to be induced by the inflammatory mediators TNF-α, IL-8 and IL-1-β in gastric epithelial cells by *Helicobacter pylori* [27] and is regulated by NF-κb [28]. Levels of IL-8 and TNF-β have recently been shown to be increased in pediatric EoE [29]. MiR146b has also been recently found to be upregulated in a murine model of asthma [11].

Increased expression of miR-21 was detected in both the pretreated EoE group as opposed to the post-treated group and in the EoE group as oppose to normal controls. MiR-21 is involved in the regulation of many different pathways and is considered an “oncomir” due to its widespread role in neoplasia. In allergic disorders miR-21 has been shown to be involved in the regulation of allergic lung inflammation by targeting IL12a (IL-12p35) a cytokine that contributes to polarization of Th cells toward Th2 cells [9]. Our finding of a six fold decrease in IL12a mRNA in this study further supports the role of IL12a as a key mediator of the allergic response [30]. MiR-21 has also been shown to play a role via TGF-β1 in pulmonary and renal fibrosis [31], [32]. One can speculate that miR-21 plays a similar role in the subepithelial fibrosis seen in EoE. MiR-21 also acts on the S100A12 protein whose gene was down regulated six fold. Seeing that S100A12 has been shown to play a key role in inflammation [33] it would be expected that S100A12 is downregulated in EoE. In addition S100A12 has also been shown to be highly expressed in differentiated esophageal epithelial cells as opposed to basal and proliferating cells [34]. EoE is associated with pronounced basal layer hyperplasia and proliferation so that the levels of S100A12 protein should be lower in EoE as opposed to normal epithelium.

MiR-203 down regulated three fold targets the ALOX15 gene (upregulated 10 fold) which encodes for arachidonate 15 lipoxygenase (15-LO). The 15-LO protein is upregulated in asthma [35] and is expressed both by eosinophils as well as airway epithelial cells [36]–[38].

As the human genome may encode over 1000 miRNAs, which may target about 60% of mammalian genes, several immune related genes which were dysregulated in our cohort had no complementary regulatory miRNA identified. Of these genes several have been shown to play a role in the pathophysiology of allergic disorders including EoE. TNFAIP6 the most significantly elevated mRNA in the pretreated biopsies as well as CPA a mast cell derived gene were both shown to be significantly upregulated in a previous study of EoE [3]. Functional network analysis in Figure 5 highlighted an NFκB-centered pathway involving both TNFAIP6 and genes in the IL-1 pathway which includes IL-RAP, IL1RN and IL1RL2. All three IL-1 related genes are down-regulated and are targets of differentially regulated miRNAs. As shown in Table 5 and 6, both targeted genes and untargeted genes contain a substantial amount of immune-related genes, indicating miRNAs and mRNAs together build sophisticated interactions contributing to the pathogenesis of EoE.

Our data implicate several miRNAs and miRNA-MRNA gene sets expressed in the epithelial layer in EoE. The identified gene expression changes provide additional diagnostic and therapeutic targets for EoE and other eosinophilic epithelial diseases.

## Supporting Information

Table S1 **The full list of 346 differentially regulated mRNAs targeted by differentially regulated miRNAs.**
(XLS)
